# Fucoxanthin Suppresses Lipid Accumulation and Inflammatory Responses in FFA-Induced Hepatocyte Models via the EGR2-CD36 Axis

**DOI:** 10.3390/molecules31142423

**Published:** 2026-07-10

**Authors:** Xiangyu Li, Chen Yang, Qionghui Chen, Xianchuan Xu, Lian Wang, Peng Zhang, Qiang Hu, Danxiang Han, Aiqun Yu, Jing Jiang, Qizhou Lian

**Affiliations:** 1 State Key Laboratory of Food Nutrition and Safety, Key Laboratory of Industrial Fermentation Microbiology of the Ministry of Education, Tianjin Key Laboratory of Industrial Microbiology, College of Biotechnology, Tianjin University of Science and Technology, Tianjin 300457, China; 2 Faculty of Synthetic Biology, Shenzhen University of Advanced Technology, Shenzhen 518107, China; 3 State Key Laboratory of Quantitative Synthetic Biology, Shenzhen Institute of Synthetic Biology, Shenzhen Institutes of Advanced Technology, Chinese Academy of Sciences, Shenzhen 518055, China; 4 State Key Laboratory of Ecological Pest Control for Fujian and Taiwan Crops, Fujian Key Laboratory of Pathogenic Fungi and Mycotoxins, School of Life Sciences, Fujian Agriculture and Forestry University, Fuzhou 350002, China; 5 Demeter Biotech Co., Ltd., Zhuhai 519075, China

**Keywords:** Fucoxanthin, MASH, CD36, lipid metabolism, bioinformatic analysis

## Abstract

Metabolic dysfunction-associated steatohepatitis (MASH) is a progressive liver disease with limited treatment options. Here, we demonstrate that fucoxanthin (FUCO), a natural marine carotenoid, attenuates free fatty acid (FFA)-induced hepatocellular steatosis and inflammatory responses in vitro by targeting the EGR2-CD36 axis (EGR2, early growth response protein 2; CD36, cluster of differentiation 36). In FFA-induced hepatocyte models (HepG2, Hep3B, and AML12), FUCO significantly reduced lipid accumulation and inflammatory markers without cytotoxicity. Mechanistic studies revealed that FUCO specifically inhibited fatty acid uptake and transport by downregulating CD36, while triglyceride (TG) degradation remained unaffected. RNA sequencing identified EGR2 as a master regulator induced by FFA and suppressed by FUCO. Functional validation showed that EGR2 overexpression completely blocked FUCO’s lipid-lowering effects and restored CD36 expression, confirming that FUCO acts through EGR2-dependent CD36 inhibition. Bioinformatic analysis further supported EGR2-mediated regulation of CD36 via tumor necrosis factor (TNF) and sterol regulatory element-binding factor (SREBF) pathways. Collectively, our findings establish EGR2 as a critical molecular target for FUCO and provide mechanistic insights that may support its further evaluation in preclinical models for MASH therapy.

## 1. Introduction

Metabolic dysfunction-associated steatotic liver disease (MASLD) has emerged as the most prevalent chronic liver disease worldwide, affecting approximately 30% of the global adult population and imposing substantial healthcare burdens [1,2]. As the progressive subtype of MASLD, metabolic dysfunction-associated steatohepatitis (MASH) is characterized by hepatic steatosis accompanied by inflammation and hepatocellular injury, which can advance to fibrosis, cirrhosis, and hepatocellular carcinoma [3,4]. Despite the urgent clinical need, therapeutic options for MASH remain severely limited. Although Resmetirom recently became the first FDA-approved drug for MASH treatment, its modest efficacy and the requirement for thyroid hormone receptor-β selectivity highlight the ongoing necessity for innovative therapeutic strategies targeting alternative molecular pathways [5,6].

The pathogenesis of MASLD is multifactorial, involving a complex interplay among genetic predisposition, nutrient excess, insulin resistance, and dysregulated lipid metabolism [7,8]. Central to disease progression is the excessive accumulation of triglycerides (TGs) in hepatocytes, driven by imbalanced lipid homeostasis encompassing increased fatty acid uptake, enhanced de novo lipogenesis, impaired β-oxidation, and reduced very-low-density lipoprotein export [9,10]. Among these, fatty acid uptake represents a critical and therapeutically targetable node. Cluster of differentiation 36 (CD36), a multifunctional scavenger receptor highly expressed in hepatocytes, serves as the primary translocase for long-chain fatty acid uptake and has been strongly implicated in MASLD pathogenesis [10,11]. Hepatic CD36 expression is markedly upregulated in MASLD patients and correlates positively with disease severity, positioning it as an attractive target for pharmacological intervention [12,13].

Natural products have garnered increasing attention as sources of novel therapeutics for metabolic diseases due to their structural diversity and favorable safety profiles. Fucoxanthin, a marine-derived carotenoid abundant in brown algae (Phaeophyceae), exhibits potent lipid-lowering, anti-inflammatory, and antioxidant activities [14,15]. Mechanistic studies have demonstrated that fucoxanthin ameliorates hepatic steatosis through multiple pathways, including upregulation of uncoupling protein 1 (UCP1) in adipose tissue, activation of AMP-activated protein kinase (AMPK), and modulation of peroxisome proliferator-activated receptor γ (PPARγ) signaling [15,16,17]. Additionally, fucoxanthin has been shown to inhibit hepatic lipogenic enzymes and promote fatty acid oxidation [15,18,19]. However, despite these advances, the precise molecular targets through which fucoxanthin exerts its hepatoprotective effects in MASH remain incompletely characterized, and its potential impact on hepatic fatty acid uptake has not been systematically investigated [20].

Early growth response protein 2 (EGR2) is a zinc finger transcription factor belonging to the immediate early gene family that plays pivotal roles in cellular stress responses, differentiation, and metabolic regulation [21]. In metabolic inflammatory environments such as obesity and MASH, macrophages internalize surplus lipids and generate lipid droplets that serve dual roles as lipid reservoirs and hubs for inflammatory mediator production [22]. Recent findings demonstrate that O-glycosylation of EGR2 at Ser299 in tumor-associated macrophages (TAMs) potentiates its transcriptional activity, driving GM-CSF-mediated pro-inflammatory macrophage differentiation [23]. This chronic inflammatory cascade accelerates lipid droplet formation in metabolic tissues (adipose and liver), creating a self-sustaining cycle between lipid dysregulation and inflammatory signaling [24]. EGR2 has been implicated in the regulation of phosphoinositide 3-kinase (PI3K)/Akt signaling [25], a critical pathway that orchestrates adipogenesis via sterol regulatory element-binding protein 1 (SREBP-1)/fatty acid synthase (FASN)-mediated lipogenesis [26]. Thus, EGR2 may indirectly participate in lipid homeostasis by acting upon the initiation stage of de novo fat production. Furthermore, EGR2 has been implicated in the development of insulin resistance and systemic metabolic dysfunction [27]. However, whether EGR2 represents a druggable target for MASH therapy and whether natural products such as fucoxanthin modulate the EGR2-CD36 axis to ameliorate hepatic steatosis remain unknown.

In this study, we employed integrated multi-omics approaches and functional genomics to systematically investigate the molecular mechanism of fucoxanthin in MASH. Using FFA-induced hepatocyte models derived from both human (HepG2 and Hep3B) and murine (AML12) origins, we demonstrate that fucoxanthin specifically suppresses fatty acid uptake and transport by downregulating CD36 expression, without affecting TG degradation pathways. Through transcriptomic profiling and bioinformatic analysis, we identify EGR2 as a master regulator of fucoxanthin’s action. Functional validation reveals that EGR2 overexpression completely abolishes fucoxanthin’s lipid-lowering effects and restores CD36 expression, establishing the EGR2-CD36 axis as the critical mechanism underlying fucoxanthin’s therapeutic efficacy. Our findings not only advance the understanding of fucoxanthin’s molecular mechanism but also position EGR2 as a novel therapeutic target for MASH, providing a mechanistic rationale for further preclinical development of fucoxanthin-based interventions.

## 2. Results

### 2.1. Effect of FUCO on Lipid Accumulation in FFA-Treated HepG2 and Hep3B Cells

**Figure 1:**
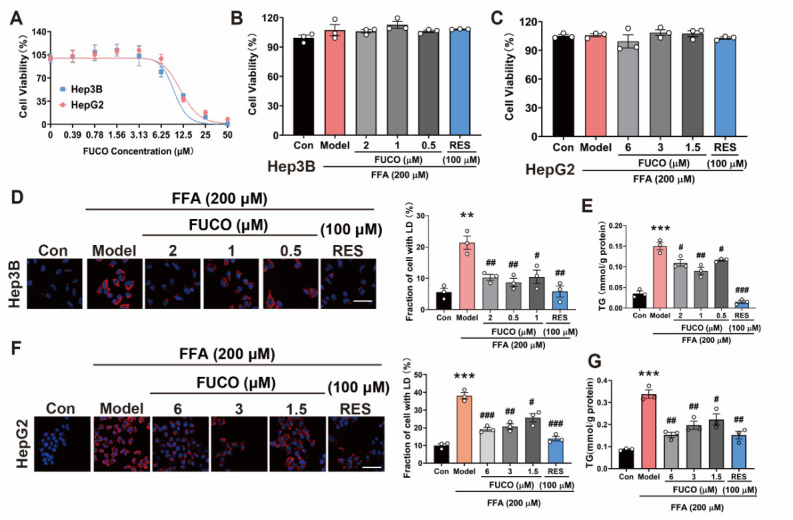
Effect of FUCO on lipid accumulation in FFA-treated HepG2 and Hep3B cells. (**A**) Cytotoxicity of fucoxanthin (FUCO) in Hep3B and HepG2 cells was assessed by CCK-8 assay following 48 h of exposure. (**B**,**C**) Cell viability of FUCO-treated Hep3B (2, 1, and 0.5 µM) and HepG2 (6, 3, and 1.5 µM) cells in the absence or presence of 200 µM of FFA for 48 h. (**D**,**F**) Lipid droplet (LD) deposition in FFA-stimulated Hep3B and HepG2 cells was assessed by Nile red staining (red); nuclei were counterstained with DAPI (blue). The fraction of LD-positive cells was quantified using Image J. Scale bar, 20 µm. (**E**,**G**) Intracellular triglyceride (TG) levels in Hep3B and HepG2 cells treated with FUCO for 12 h, followed by co-treatment with FFA and FUCO for 24 h. Experiments were performed in triplicate, and each value represents the mean ± SEM. ** *p* < 0.01, and *** *p* < 0.001 vs. the control; ^#^ *p* < 0.05, ^##^ *p* < 0.01, and ^###^ *p* < 0.001 vs. the model.

To determine the non-toxic concentration range, hepatocyte viability was assessed by CCK-8 assay following 48 h of FUCO exposure (Figure 1A), as well as following 48 h of Resmetirom treatment (Figure S1A). To evaluate the therapeutic potential of FUCO against MASH, we established an FFA-induced Hep3B cell model. Cell viability was first confirmed in FUCO-treated Hep3B (Figure 1B) and HepG2 (Figure 1C) cells with or without FFA co-treatment, validating the safety of selected concentrations. Nile red staining revealed that FUCO significantly reduced lipid accumulation in FFA-treated cells in a dose-dependent manner (Figure 1D,F). This finding was corroborated by TG quantification in both cell lines (Figure 1E,G). Together, these results demonstrate that FUCO effectively attenuates FFA-induced lipid accumulation in hepatocytes.

### 2.2. Effect of FUCO on Lipid Accumulation in Murine Hepatocytes

**Figure 2:**
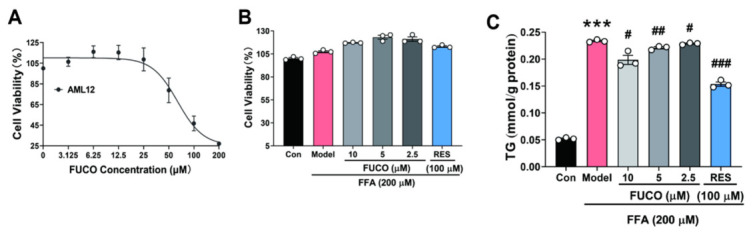
Effect of FUCO on lipid accumulation in murine hepatocytes. (**A**) Cytotoxicity of FUCO in AML12 cells was assessed by CCK-8 assay following 48 h of exposure. (**B**) Cell viability of AML12 cells treated with FUCO (10, 5, and 2.5 µM) in the absence or presence of 200 µM of FFA for 48 h. (**C**) TG level in AML12 cells treated with FUCO for 12 h, followed by co-treatment with FFA and FUCO for 24 h. Experiments were performed in triplicate, and each value represents the mean ± SEM. *** *p* < 0.001 vs. the control; ^#^ *p* < 0.05, ^##^ *p* < 0.01, and ^###^ *p* < 0.001 vs. the model.

To further evaluate the therapeutic efficacy of FUCO against MASH, we first assessed the viability of AML12 cells (a normal murine hepatocyte line) using the CCK-8 assay to determine non-toxic concentrations (Figure 2A). Cell viability was subsequently re-assessed in FUCO-treated AML12 cells with or without FFA exposure, confirming the safety of selected doses for subsequent experiments (Figure 2B). The lipid-lowering efficacy of FUCO was further validated by measuring TG levels in these cells (Figure 2C). Collectively, these findings demonstrate that FUCO exerts a direct protective effect on murine hepatocytes.

### 2.3. FUCO Alleviates FFA-Induced Steatosis and Inflammatory Responses in Hepatocytes and THLE2 Cells

**Figure 3:**
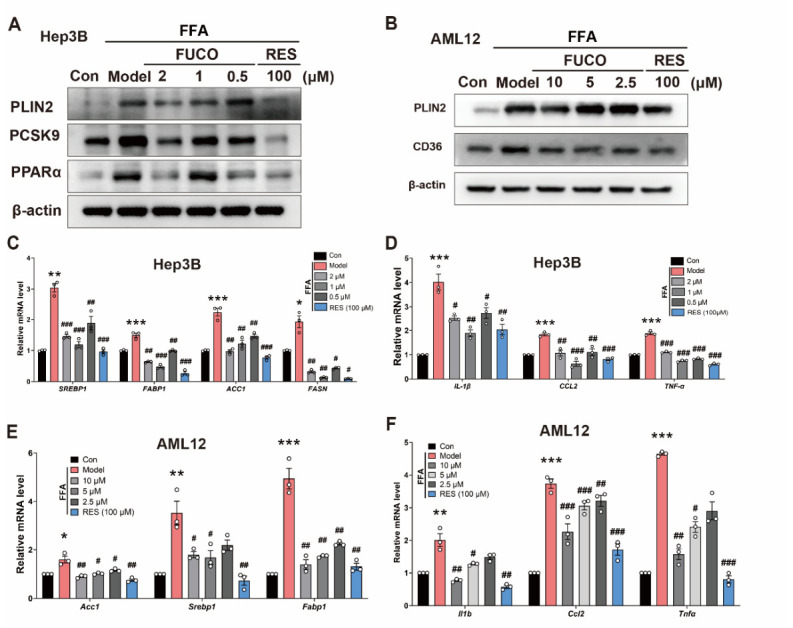
FUCO attenuates the cellular model of steatosis in FFA-treated hepatocytes through suppression of inflammation. (**A**–**D**) Expression of lipid-metabolizing enzymes at protein (Western blot, (**A**,**B**)) and mRNA (qRT-PCR, (**C**,**D**)) levels in Hep3B (**A**,**C**) and AML12 (**B**,**D**) cells. (**E**,**F**) Inflammatory marker mRNA levels in Hep3B (**E**) and AML12 (**F**) cells. Cells were pretreated with FUCO (12 h) prior to FFA and FUCO co-treatment (24 h). Experiments were performed in triplicate, and each value represents the mean ± SEM. * *p* < 0.05, ** *p* < 0.01, and *** *p* < 0.001 vs. the control; ^#^ *p* < 0.05, ^##^ *p* < 0.01, and ^###^ *p* < 0.001 vs. the model.

Although fucoxanthin has demonstrated hepatoprotective effects in preclinical studies [28,29] and has shown therapeutic promise against MASLD in both human [19,30] and animal models [28,31], its underlying mechanism of action remains to be fully clarified. Here, we showed that FUCO attenuated FFA-induced hepatocellular steatosis and inflammatory responses in Hep3B and AML12 cells by modulating the expression of lipid metabolism-related genes and proteins at both transcriptional and protein levels (Figure 3A–D). In parallel, FUCO significantly reduced the expression of inflammatory markers in FFA-treated Hep3B and AML12 cells after 24 h of treatment (Figure 3E,F), indicating that FUCO alleviates both lipid accumulation and inflammatory responses in hepatocytes.

To address the concern that these effects might be specific to hepatocellular carcinoma-derived cell lines, we further validated FUCO in THLE2 cells, a non-tumorigenic human hepatic cell line. As shown in Figure S2, FUCO reduced the FFA-induced mRNA expression of lipogenic genes, including ACC1, FASN, and CD36, suppressed the inflammatory genes CCL2 and IL-6, and decreased the protein levels of PLIN2, PCSK9, and SREBP1. These findings support the notion that FUCO exerts similar protective effects in a more physiologically relevant hepatic model.

### 2.4. FUCO Inhibits the Uptake and Transport of FFA in Hepatocytes

**Figure 4:**
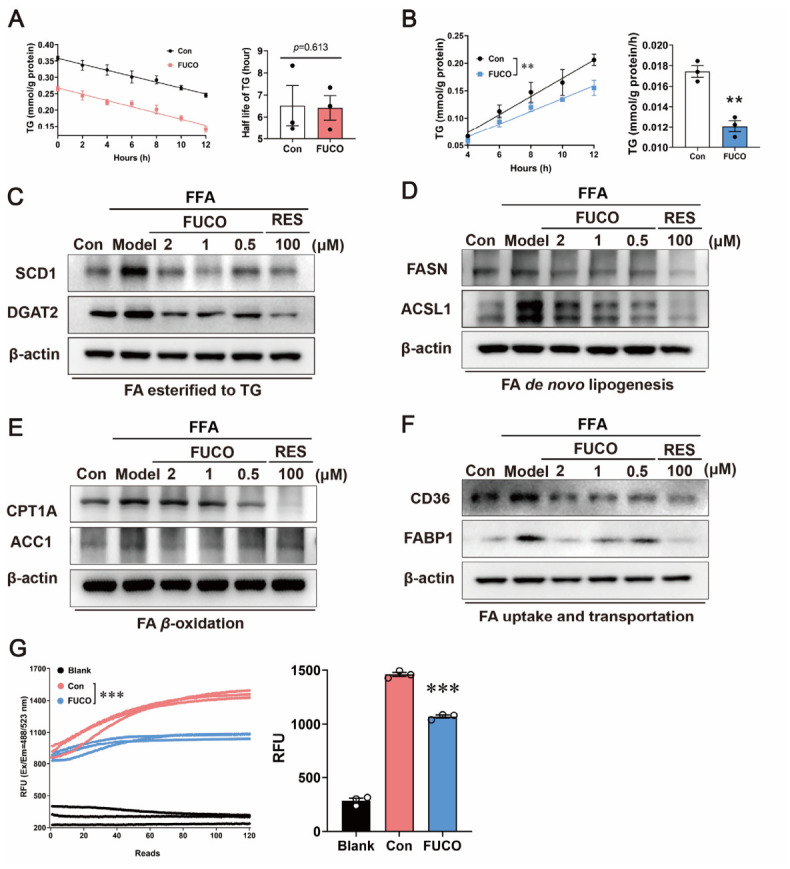
FUCO inhibits the uptake and transport of FFA in hepatocytes. (**A**,**B**) Half-life (**A**) and synthesis rate (**B**) of TG in Hep3B cells. (**C**–**F**) Protein levels detected by Western blot associated with FA esterified to TG (**C**), *de novo* lipogenesis (**D**), beta-oxidation (**E**), and uptake and transport (**F**) in Hep3B cells treated with FUCO and FFA. (**G**) FFA uptake and transport in Hep3B cells treated with FUCO. Experiments were performed in triplicate, and each value represents the mean ± SEM. ** *p* < 0.01, and *** *p* < 0.001 vs. the control.

Given the regulatory role of FUCO in hepatic lipid metabolism, we investigated its impact on intracellular lipid droplet accumulation. First, we determined the specific stage of lipid droplet accumulation targeted by FUCO. Hep3B cells were pretreated with FUCO for 12 h, followed by FFA stimulation for 24 h. Intracellular TG levels were measured following cycloheximide (CHX) treatment at a concentration determined by CCK-8 assay (Figure S1B). No significant difference in TG half-life was observed between the FUCO-treated and control groups (Figure 4A), implying that fucoxanthin does not modulate intracellular TG degradation. Next, we investigated the impact of FUCO on TG synthesis. Intracellular TG levels were measured following tyloxapol treatment, a lipoprotein lipase inhibitor that binds to lipase to block triglyceride hydrolysis, at a concentration determined by CCK-8 assay (Figure S1B) [32]. Fucoxanthin treatment significantly lowered TG synthesis rates versus the control group (Figure 4B), demonstrating its inhibitory effect on intracellular TG synthesis.

The process of TG synthesis encompasses four stages: fatty acid uptake and transport, β-oxidation, de novo lipogenesis, and final esterification to TG by enzymes localized in the endoplasmic reticulum [33,34]. We thus explored which TG synthesis stage FUCO influenced. FUCO and FFA were treated in Hep3B cells. The protein levels that involved the four stages of TG synthesis showed that the levels of protein involved with FA esterified to TG (such as the endoplasmic reticulum-bound stearoyl-CoA desaturase 1 (SCD1) and diacylglycerol acyltransferase 2 (DGAT2)) (Figure 4C), with FA de novo lipogenesis (such as long-chain fatty-acid-coenzyme A ligase 1 (ACSL1), fatty acid synthase (FASN)) (Figure 4D), FA β-oxidation (such as carnitine Palmitoyl transferase 1 alpha (CPT1A), and acetyl Co-A carboxylase 1 (ACC1)) (Figure 4E), and FA uptake and transport (such as fatty acid binding protein 1 (FABP1), with cluster of differentiation 36 (CD36)) decreased in the FUCO-treated Hep3B cells in dose-dependent manners.

Similarly, CD36, along with TG deposition-related proteins, such as PLIN2 (Figure 3B), were decreased in AML12 cells with FFA- and FUCO-treated groups compared to the control, suggesting that FUCO decreases the level of CD36 protein and thus may influence TG synthesis by inhibiting FA uptake and transport. Indeed, examination of FA uptake and transport discovered that FUCO treatment in Hep3B cells led to a significant reduction in FA absorption (Figure 4G), and the consequences may cause a decrease in the protein levels of enzymes involved with FA β-oxidation (Figure 4E), FA de novo lipogenesis (Figure 4D), and FA esterified to TG (Figure 4C), which are the TG synthesis stages after FA uptake and transport. These results collectively indicate that downregulation of CD36 by FUCO may lead to inhibition of the uptake and transport of FA.

### 2.5. FUCO Attenuates the Cellular Model of Steatosis in FFA-Treated Hepatocytes Through Downregulating EGR2

**Figure 5:**
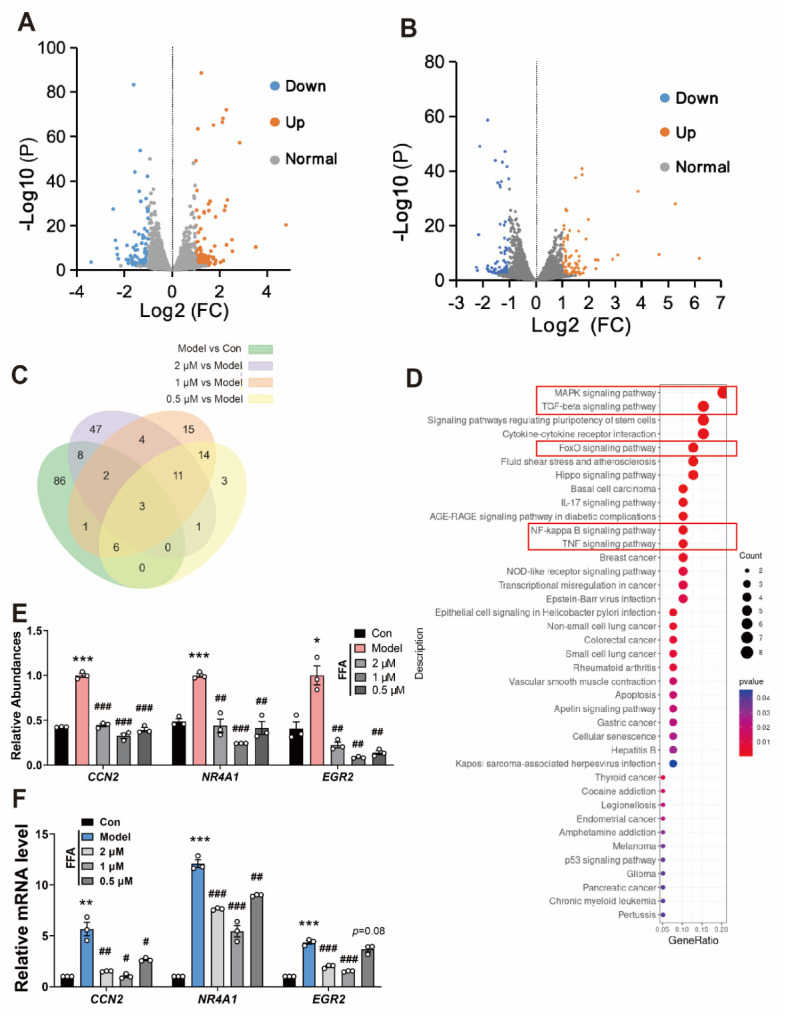
FUCO attenuates the cellular model of steatosis in FFA-treated hepatocytes through downregulation of EGR2. (**A**,**B**) Volcanic plots showing the analysis of different genes. (**C**) Venn diagram verifying different genes among groups. (**D**) KEGG signaling pathway analysis of differential genes. (**E**) Relative abundances of different genes. (**F**) mRNA levels quantified by qRT-PCR in Hep3B cells. The experiments were performed in triplicate, and each value represents the mean ± SEM. * *p* < 0.05, ** *p* < 0.01, and *** *p* < 0.001 vs. the control; ^#^ *p* < 0.05, ^##^ *p* < 0.01, and ^###^ *p* < 0.001 vs. the model.

To elucidate the molecular mechanisms underlying the anti-steatotic effects of FUCO, we performed RNA sequencing (RNA-seq) analysis to compare the transcriptomic profiles of the control, FFA-induced steatosis model, and FUCO-treated groups. As shown in Figure 5A, 106 mRNAs were differentially expressed in the livers of the model and control groups, and 76 were differentially expressed between the FUCO-1 and model groups (Figure 5B). A Venn diagram was used to summarize the differentially expressed mRNAs among groups, and three overlapping mRNAs were identified (Figure 5C). As shown in Figure 5D,E, the differentially expressed genes of FUCO are highly involved in the MAPK signaling pathway and TGFβ signaling pathway, indicating that FUCO may alleviate fatty acid-induced lipid deposition and liver inflammation through EGR2. Subsequently, qRT-PCR was performed to determine hepatocyte EGR2 expression in each group. EGR2 expression was dramatically increased in the model group, and FUCO-1 treatment significantly reversed this increase (Figure 5F), which was consistent with the detected data (Figure 5E). Therefore, EGR2 may be an important target of FUCO to improve lipid droplet deposition and inflammation in hepatocytes.

### 2.6. FUCO Suppresses Fatty Acid Uptake via EGR2-Mediated Downregulation of CD36

**Figure 6:**
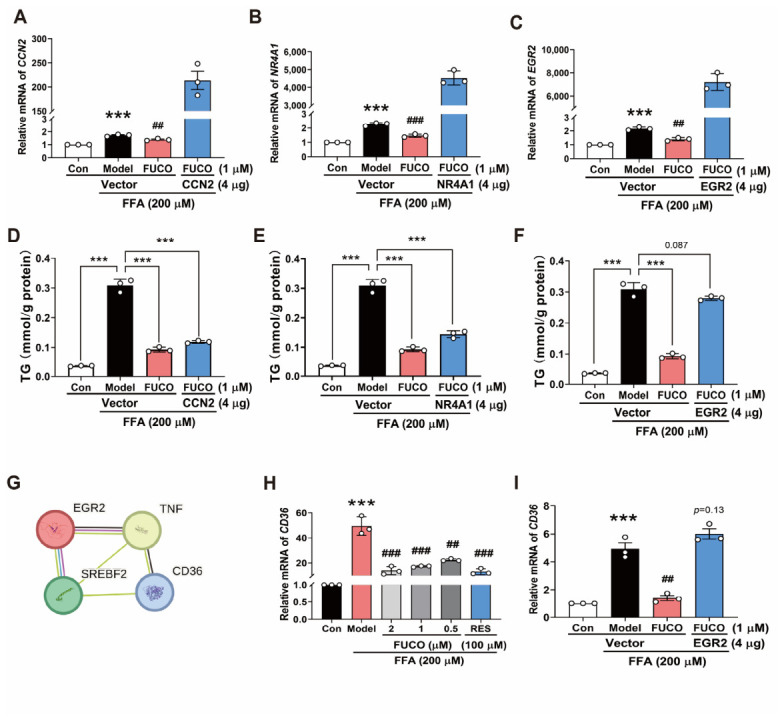
FUCO suppresses fatty acid uptake via EGR2-mediated downregulation of CD36. (**A**–**C**) RNA levels in Hep3B cells transfected with CCN2 (**A**), NR4A1 (**B**), and EGR2 (**C**) plasmid for 12 h and then treated with FFA for 24 h prior to treatment with FUCO for 12 h. (**D**–**F**) Intracellular TG levels in Hep3B cells transfected with CCN2 (**D**), NR4A1 (**E**), and EGR2 (**F**) plasmid for 12 h and then treated with FFA for 24 h prior to treatment with FUCO for 12 h. (**G**) The regulation of CD36 by EGR2 predicted in the STRING database. (**H**) CD36 mRNA levels treated with FUCO in Hep3B cells. (**I**) CD36 mRNA levels transfected with EGR2 in Hep3B cells. The experiments were performed in triplicate, and each value represents the mean ± SEM. *** *p* < 0.001 vs. the control; ^##^ *p* < 0.01, and ^###^ *p* < 0.001 vs. the model.

We next sought to elucidate the molecular mechanisms underlying the protective effects of FUCO against MASH-related cellular phenotypes. Hep3B cells were transfected with overexpression plasmids for CCN2, NR4A1, or EGR2 and subsequently treated with FFA. Intracellular TG levels were significantly reduced in the empty vector group upon FUCO treatment. In contrast, TG levels in cells overexpressing CCN2 (Figure 6A,D) or NR4A1 (Figure 6B,E) remained comparable to those in the empty vector plus FUCO cotreatment group, indicating that the lipid-lowering effect of FUCO is not primarily mediated by CCN2 or NR4A1. Notably, EGR2 overexpression markedly attenuated the lipid-lowering effect of FUCO (Figure 6C,F), suggesting that EGR2 may be involved in FUCO-mediated suppression of lipid accumulation.

To further validate the role of EGR2 in FUCO action, we performed EGR2 loss-of-function experiments in THLE2 cells using three independent siRNAs. Among these, siEGR2-1 and siEGR2-3 efficiently reduced EGR2 mRNA expression, with siEGR2-1 showing the strongest silencing effect (Figure S3A). Consistent with the overexpression data, EGR2 knockdown significantly decreased intracellular TG content and CD36 mRNA expression (Figure S3B,C). Importantly, in EGR2-silenced cells, FUCO did not further reduce TG accumulation or CD36 expression to a statistically significant extent (TG, *p* = 0.72; CD36, *p* = 0.23), indicating that the inhibitory effect of FUCO on lipid accumulation and CD36 expression is attenuated when EGR2 is suppressed.

To explore the downstream mechanisms by which EGR2 regulates fatty acid metabolism, we performed STRING database analysis [35], which predicted that EGR2 may regulate CD36 through TNF and SREBF (Figure 6G). As shown in Figure 3B and Figure 4F,G, FUCO reduces fatty acid uptake and transport by inhibiting CD36 expression. Together, these findings support a model in which FUCO suppresses CD36 expression and reduces fatty acid uptake and transport, at least in part, through an EGR2-dependent mechanism. Thus, EGR2 emerges as an important mediator of FUCO action in the attenuation of FFA-induced hepatocellular lipid accumulation.

## 3. Materials and Methods

### 3.1. Cell Culture

The human hepatocyte HepG2 cells were obtained from the cryopreserved cell repository maintained in our laboratory [36]. The human hepatocyte Hep3B (CL-0102, Wuhan, China) and THLE2 (CL-0833, Wuhan, China) cells were purchased from Procell. The AML12 (alpha mouse liver 12) cells were kindly provided by Professor Er-fei Song (Institute of Metabolic Science, Science and Technology Innovation Center, Guangzhou University of Chinese Medicine, Guangzhou, China). HepG2 was cultured in Modified Eagle Medium (MEM) (12571063, Gibco, Grand Island, NY, USA) containing 10% fetal bovine serum (A5256701, Gibco, USA) and 1% penicillin/streptomycin (P/S) (15140122, Gibco, USA). Hep3B cells were cultured with MEM, 10% FBS, 1% non-essential Amino Acid (NEAA) (C0332, beyotime, Shanghai, China), and 1% P/S. AML12 cells were cultured in AML12 Cell Complete Medium (CM-0602, Procell, Wuhan, China). THLE2 cells were cultured in THLE2 Cell Complete Medium (CM-0833, Procell, Wuhan, China). All cell lines were cultured at 37 °C with 5% CO_2_.

### 3.2. Cytotoxicity Assay

The cytotoxicity of fucoxanthin (FUCO, Demeter biotech, Zhuhai, China), cycloheximide (CHX, T1225, TagerMol, Shanghai, China), and tyloxapol (TLP, T0307, TagerMol, China) in cells was assayed using the cell counting kit (CCK-8, K1018, Apexbio, Houston, TX, USA) staining method [37]. In brief, HepG2, Hep3B, or AML12 cells (1 × 10^4^ cells per well) were seeded in 96-well plates overnight at 37 °C under 5% CO_2_. The media was removed, and serial two-fold dilutions of FUCO, CHX, Resmetirom, or TLP were applied for 48 h. After incubation at the corresponding time, 10 μL of CCK-8 was added to each well and incubated at 37 °C for 1–2 h. The solution absorbance was measured at 450 nm using BioTek Synergy HTX (S1LFA-SN, Agilent, Santa Clara, CA, USA). Resmetirom (RES, T3595, TagerMol, Shanghai, China) served as the positive control in all pharmacological testing experiments.

### 3.3. Nile Red Staining

HepG2 or Hep3B cells were plated on cell-climbing slices (YA0350, Solarbio, Beijing China) pre-coated with rat tail collagen in 6-well plates and then treated with 200 µM of FFA for 24 h. The FFA mixture consisted of oleic acid (OA) and palmitic acid (PA) at a 2:1 molar ratio (8 mM OA and 4 mM PA), which was dissolved in 10% fatty acid-free bovine serum albumin (BSA) to facilitate fatty acid uptake. Then, the cells were fixed with 3% paraformaldehyde and permeabilized with 0.5% Triton X-100 (ST1723, Beyotime, China). Nile Red (19123, sigma, St. Louis, MO, USA) solution at 10 μM was added to incubate for 10 min. Then, the cells were washed with PBS (BL302A, Biosharp, Hefei, China), sealed by mounting the medium supplied with DAPI (ZLI-9557, ZSGB-BIO, Beijing, China) staining solution, and dried in the dark. The images were acquired using a confocal microscope (FV4000, Olympus, Tokyo, Japan) at a magnification of 40×. The fraction of cells with LD was quantified by image J.

### 3.4. RNA Sequencing and Analysis

Total RNA was extracted from Hep3B cells, and 1 μg per sample was used for mRNA library preparation with polyA enrichment. Three biological replicates per condition were sequenced on an Illumina NovaSeq X Plus/DNBSEQ T7 platform with paired-end 150 bp reads, generating an average of 15 million raw reads per sample. The quality of raw sequencing reads was verified using Fastp for quality control, adapter trimming, filtering, and base correction. Clean reads were aligned to the *Homo sapiens* reference genome GRCh38.p14 using STAR version 2.7.11b, with an average of 71% of clean reads uniquely mapped to the reference genome; the relatively lower assignment rate is attributable to the inclusion of comprehensive non-coding annotations in GENCODE v46. Gene-level quantification was performed using featureCounts from the R package Rsubread version 2.16.1. All samples originated from a single sequencing run, and principal component analysis revealed no evidence of batch effects; therefore, no batch correction was applied. Normalization, differential expression analysis, and principal component analysis were performed using the R package DESeq2 v1.42.1. Specifically, raw counts were internally normalized by the median-of-ratios method to account for differences in library size and RNA composition, and count data were transformed using regularized logarithm (rlog, blind = FALSE) for downstream visualization. Differentially expressed genes (DEGs) were identified using the Wald test with Benjamini–Hochberg false discovery rate correction; genes with an adjusted *p*-value < 0.05 and |log_2_ fold change| > 1 were considered significant. KEGG pathway enrichment analysis of DEGs was performed using the clusterProfiler R package version 4.10.1, with gene ID conversion via the org.Hs.eg.db database, pathway enrichment based on the KEGG database, and visualization of enriched pathways using the ggplot2 plotting system. The RNA-seq data have been deposited in the GEO database under accession number GSE326385 (NCBI tracking system #25711093).

### 3.5. Quantitative Real-Time RT-PCR Analyses

Total RNA in cells was extracted using SteadyPure Universal RNA Extraction Kit II (AG21022, ACCURATE BIOLOGY, Changsha, China), according to the manufacturers’ instructions. Total RNA quality and concentrations were determined using a Nanodrop one (NanoDrop Inc., Wilmington, DE, USA). The RNA was quantified with one-step real-time quantitative reverse transcript PCR (qRT-PCR) using Evo M-MLV One Step RT-qPCR Kit (SYBR, AG11732, ACCURATE BIOLOGY, China). The expression of GAPDH was used as an internal control. Primer sequences are listed in Table S1.

### 3.6. Plasmid Construction and Transfection Assay

The full-length of CCN2 and NR4A1 were generated using standard PCR methods and primers (Table S2) and then subcloned into the corresponding pLV-CMV vectors. EGR2 plasmid was purchased from MIAOLING PLASMID (P94876, Wuhan, China). All plasmids were confirmed by sequencing. For overexpression of human protein, the plasmid was transfected into cells with PEI MAX^®^ Transfection Reagent (MW 40,000, 24765, polysciences, Warrington, PA, USA) in Opti-MEM (11058021, Gibco, Grand Island, NY, USA). After transfection of 48 h, intracellular proteins were detected by Western blot assay.

### 3.7. siRNA Transfection and FUCO Treatment

Three independent small interfering RNAs (siRNAs) targeting human EGR2 (siEGR2-1, siEGR2-2, and siEGR2-3) and a negative control siRNA (NC) were synthesized by Primerna Biochem Co., Ltd. (Shanghai, China). THLE2 cells were seeded in 6-well plates and transfected with NC or EGR2-specific siRNAs using Lipofectamine 2000 reagent (Mei5bio, MF135-01, Beijing, China), according to the manufacturer’s instructions. Briefly, cells were transfected with 50 pmol of siRNA per well and incubated for 6 h. The transfection medium was then replaced with complete medium either with or without FUCO (10 μM), and the cells were further cultured for 30 h. After treatment, cells were harvested for RNA extraction and intracellular triglyceride (TG) analysis.

### 3.8. TG Turnover and Secretion Assay

TG turnover assay was tested following the method previously described [38,39]. Briefly, Hep3B cells were treated with or without 1 µM of FUCO incubated for 12 h in based medium. Then, the cells were induced by 200 µM of FFA with or without 1 µM of FUCO. After 24 h, the medium was replaced with regular growth media containing 100 µg/mL of CHX. Intracellular TGs were extracted at designated times as indicated in the figure legends and quantified using detection kits (E1013, Applygen, Beijing, China). The protein contents in the cells were quantified using the Enhanced BCA Protein Assay Kit (P0010, Beyotime, Shanghai, China). The half-time of TGs was calculated based on the fitting curve. Similarly, we used the Hep3B cells to evaluate the TG secretion. After treatment with 1 µM of FUCO for 36 h, the cells were induced by 200 µM of FFA containing 50 µM of TLP. Intracellular TGs and protein contents were also detected to calculate the hepatic TG secretion based on the fitting curve.

### 3.9. Fatty Acid Uptake Assay

Fatty acid uptake activities were assessed using the Free Fatty Acid Uptake Assay Kit (ab287857, Abcam, Cambridge, UK), following the manufacturer’s instructions. Briefly, Hep3B cells were seeded in a 96-well plate, and treated with or without 10 µM of FUCO for 30 min. After washing with PBS, the cells were incubated in a serum-free medium for one hour, followed by treatment with a fluorescent fatty acid mixture for 30 min. Fluorescence intensity was measured using a microplate fluorescence reader at 485 nm/528 nm. The fluorescence signal of the control group was used as the baseline for relative quantification.

### 3.10. Western Blot Analyses

Total proteins from the mouse liver tissues and cells were, respectively, extracted using Protein Extraction Reagent (20118ES60, Yesen, Shanghai, China) supplemented with a protease and phosphatase inhibitor (P1045, Beyotime, Shanghai, China). Total protein content was quantified using the Enhanced BCA Protein Assay Kit. Protein samples were separated on 7.5% to 12.5% concentration SDS-PAGE gels (20325ES62, Yesen, Shanghai, China) and transferred onto PVDF membranes (IPVH00010; Millipore, Billerica, MA, USA). The membranes were then blocked with 5% non-fat powdered milk (M33421, Mei5bio, Beijing, China) in 1 × TBST (60145ES76, Yesen, Shanghai, China), and then incubated overnight at 4 °C with the respective primary antibodies listed in Table S3. After being washed with TBST, the membranes were incubated with HRP-conjugated secondary antibodies (AS014, ABclonal, Wuhan, China) for 1 h at room temperature. Protein expression signals were visualized using GelView 6000Plus (Biolight Biotechnology, Guangzhou, China), with β-actin as the internal control. The primary antibodies used in this study, along with their sources, catalog numbers, and dilution ratios, are listed in Table S3 in the Supplementary Materials.

### 3.11. Ethics Statement

This study used commercially available cell lines and did not involve any human participants, human tissues, or animal experiments. Therefore, no specific ethical approval was required.

### 3.12. Statistical Analyses

The data were presented as the mean ± Standard Error of the Mean (SEM) and representative figures. Statistical analysis was performed using SPSS17.0 or GraphPad Prism 8 (San Diego, CA, USA) and analyzed by Student’s *t*-test or analysis of variance (ANOVA) followed by Student–Newman–Keuls (SNK) post hoc tests. The value of statistical significance was set as *p* < 0.05.

## 4. Discussion

**Figure 7:**
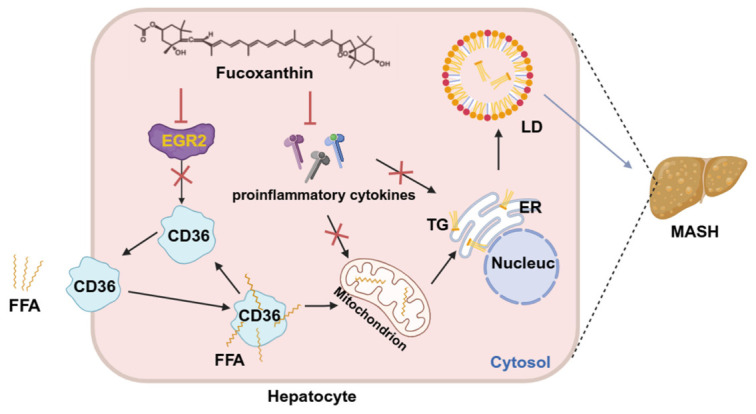
A schematic diagram depicting the mechanism of fucoxanthin in hepatic steatosis. Fucoxanthin suppresses EGR2 expression, thereby inhibiting CD36-mediated fatty acid uptake and transport in hepatocytes, consequently reducing intracellular triglyceride accumulation and ameliorating hepatic steatosis. Furthermore, fucoxanthin attenuates the production of pro-inflammatory cytokines, providing additional protection against MASH progression. FFA, free fatty acid; EGR2, early growth response protein 2; CD36, cluster of differentiation 36; TG, triglyceride; ER, endoplasmic reticulum; LD, lipid droplet; and MASH, metabolic dysfunction-associated steatohepatitis.

Metabolic dysfunction-associated steatotic liver disease (MASLD) is the most prevalent chronic liver disease globally, and its progressive form, MASH [40,41,42,43], remains therapeutically challenging despite the recent FDA approval of Resmetirom [5]. Fucoxanthin, a marine-derived carotenoid, has garnered attention for its lipid-lowering properties [44], but its precise molecular targets in MASH remain incompletely characterized. In this study, we used FFA-treated hepatocyte cell lines (HepG2, Hep3B, and AML12) as in vitro models of hepatocellular steatosis and lipid-induced inflammatory responses. While these models allow for mechanistic dissection of early lipid accumulation and selected inflammatory pathways, they do not recapitulate the full histopathological spectrum of MASH, which includes hepatocellular ballooning and fibrosis. Therefore, our conclusions are limited to the in vitro context and await validation in appropriate animal models. In this study, we provide compelling evidence that fucoxanthin suppresses FFA-induced hepatocellular lipid accumulation and inflammatory responses primarily through suppression of the EGR2-CD36 axis, thereby inhibiting fatty acid uptake and subsequent lipid accumulation (Figure 7).

Our investigation revealed that fucoxanthin treatment significantly reduced FFA-induced lipid accumulation in both human (HepG2, Hep3B) and murine (AML12) hepatocytes, without compromising cell viability. Notably, the reduction in hepatic TG content was not accompanied by compensatory inflammation, suggesting that fucoxanthin confers genuine protective effects rather than merely redistributing lipids. Notably, the lipid-lowering effect of FUCO appeared more pronounced in human hepatocyte-derived cell lines (HepG2 and Hep3B) compared to murine AML12 cells, even at lower concentrations. This differential sensitivity may reflect species-specific differences in fatty acid metabolism, CD36 expression and regulation, or the bioavailability of FUCO in hepatocytes of different origins. Additionally, the transformed nature of HepG2 and Hep3B cells, as opposed to the non-transformed phenotype of AML12 cells, may contribute to distinct metabolic and drug-response profiles. Future comparative studies using primary human and murine hepatocytes would be valuable to further clarify these species-specific effects. Mechanistic dissection demonstrated that fucoxanthin specifically targeted the fatty acid uptake and transport stage of TG synthesis, as evidenced by unchanged TG half-life but significantly reduced fatty acid absorption. This selective inhibition was mediated by downregulation of CD36, a key fatty acid translocase highly expressed in hepatocytes and implicated in MASLD pathogenesis [45,46].

The identification of EGR2 as the upstream regulator of fucoxanthin’s action represents a significant finding of this study. EGR2, a zinc finger transcription factor of the early growth response family, has been increasingly recognized as a critical mediator of metabolic homeostasis. Our transcriptomic analysis revealed dramatic EGR2 induction in FFA-treated hepatocytes, which was effectively reversed by fucoxanthin treatment. Functional validation through overexpression studies unequivocally established EGR2 as an essential mediator of fucoxanthin’s effects: while CCN2 and NR4A1 overexpression failed to modulate fucoxanthin’s lipid-lowering activity, EGR2 overexpression completely abolished its therapeutic efficacy. These findings position EGR2 as a novel and critical target for fucoxanthin in the context of MASH.

The Venn diagram analysis revealed a limited set of overlapping differentially expressed genes across the experimental groups. Among these, EGR2 emerged as a particularly significant target, as it was consistently upregulated by FFA stimulation and effectively suppressed by FUCO treatment. The relatively small number of overlapping genes likely reflects the fact that FUCO exerts its effects through targeted modulation of key regulatory nodes (such as EGR2) rather than through broad, non-specific transcriptional changes. Furthermore, the non-overlapping differentially expressed genes were significantly enriched in pathways critical to MASH pathogenesis, including MAPK signaling and TGFβ signaling, suggesting that FUCO may also influence additional signaling cascades beyond the EGR2-CD36 axis. Collectively, these findings support the model that FUCO acts through a focused but mechanistically coherent transcriptional program to alleviate FFA-induced lipid accumulation and inflammation.

The mechanistic link between EGR2 and CD36 was supported by bioinformatic analysis using the STRING database, which predicted regulatory connections through TNF and SREBF pathways. Experimentally, we demonstrated that fucoxanthin-mediated CD36 suppression was completely abrogated in EGR2-overexpressing cells, paralleling the loss of lipid-lowering effects. This establishes a coherent mechanistic framework wherein fucoxanthin suppresses EGR2 expression, thereby relieving EGR2-mediated CD36 suppression transcription and consequently reducing fatty acid uptake and hepatic lipid accumulation. This EGR2-CD36 axis represents a previously unrecognized mechanism of fucoxanthin action that may be therapeutically exploitable.

From a translational perspective, our findings carry significant implications. First, the identification of EGR2 as a molecular target provides a pharmacodynamic biomarker for monitoring fucoxanthin efficacy in clinical settings. Second, the demonstration that fucoxanthin acts through a specific molecular axis rather than pleiotropic effects supports its development as a mechanism-based therapeutic agent. Third, given the central role of CD36 in MASLD pathogenesis and its emergence as a therapeutic target [47,48], fucoxanthin represents a natural product-based approach to CD36 modulation with potentially favorable safety profiles compared to synthetic inhibitors [12,13].

Several limitations of this study should be acknowledged. First, our investigations were conducted exclusively in cell-based models, and validation in animal models of MASH and ultimately in human clinical trials will be essential. Second, while our loss-of-function and gain-of-function experiments provide strong genetic evidence supporting an EGR2-CD36 regulatory axis, they do not formally establish direct transcriptional regulation of CD36 by EGR2. The STRING-based bioinformatic predictions involving TNF and SREBF pathways are hypothesis-generating and require further validation using ChIP-qPCR, CD36 promoter luciferase reporter assays, and mutagenesis of putative EGR2 binding sites. Third, we acknowledge that FFA-treated hepatocyte models recapitulate hepatocellular lipid loading and selected inflammatory responses but do not reproduce the full histopathological spectrum of MASH, which requires steatosis, hepatocellular ballooning, inflammation, and fibrosis assessment. Fourth, the precise molecular mechanism by which fucoxanthin downregulates EGR2 expression—whether through direct transcriptional inhibition, modulation of upstream signaling pathways such as MAPK or TGFβ, or epigenetic mechanisms—requires further investigation.

In conclusion, this study establishes fucoxanthin as a promising candidate for further preclinical evaluation against MASH through its novel action on the EGR2-CD36 axis. By suppressing EGR2-mediated CD36 suppression, fucoxanthin specifically inhibits fatty acid uptake, thereby ameliorating hepatic steatosis and inflammation without provoking compensatory metabolic disturbances. These findings not only advance our understanding of fucoxanthin’s molecular mechanism but also highlight EGR2 as a potential therapeutic target for MASH. Future studies should focus on validating this mechanism in preclinical animal models and exploring the translational potential of fucoxanthin-based interventions for MASH patients.

## Data Availability

Data will be made available upon request.

## Supplementary Materials

The following supporting information can be downloaded at: https://www.mdpi.com/article/10.3390/molecules31142423/s1, Figure S1: Cytotoxic effects of RES, TLP and CHX on different liver cell lines; Figure S2: FUCO attenuates lipid deposition in FFA-treated THLE2 cells by inhibiting inflammation; Figure S3: EGR2 is involved in FUCO-mediated suppression of CD36 expression and lipid accumulation in THLE2 cells; Table S1: qPCR Primer and siRNA sequence; Table S2: Primers for plasmid construction; Table S3: Antibody.
