# Supplementary material for: Fucoxanthin Suppresses Lipid Accumulation and Inflammatory Responses in FFA-Induced Hepatocyte Models via the EGR2-CD36 Axis
Source: Molecules. 2026 Jul 10;31(14):2423. doi: 10.3390/molecules31142423 (PMC13414028; doi:10.3390/molecules31142423)
Supplement: Supplementary file 1 [file molecules-31-02423-s001.zip › molecules-4278738-supplementary-proofreading.pdf]

Fucoxanthin suppresses lipid accumulation and inflammatory responses in FFA-induced hepatocyte models via the EGR2-CD36 axis

1. Materials and methods

Table S1 qPCR Primer

GENE		Primer（5'-3'）
Human *SREBP1*	Forward	TACCACCAGCGTCTACCATAGCC
	Reverse	CTTGCGATGCCTCCAGAAGTACAC
Human *FASN*	Forward	ACAGCGGGGAATGGGTACT
	Reverse	GACTGGTACAACGAGCGGAT
Human *FABP1*	Forward	TCGGAAATCGTGCAGAATGGGAAG
	Reverse	TGGTGATTATGTCGCCGTTGAGTTC
Human *ACC1*	Forward	TGTCCTTCTCCTCCAACCTCAACC
	Reverse	CTGCCAGCCTGTCATCCTCAATATC
Human *CD36*	Forward	GGCTGTCATTGGTGCTGTCCTG
	Reverse	TGCTGCTGTTCATCATCACTTCCTG
Human *CCL-2*	Forward	CAGCCAGATGCAATCAATGCC
	Reverse	TGGAATCCTGAACCCACTTCT
Human *TNF-α*	Forward	CAAGGACAGCAGAGGACCAG
	Reverse	TGGCGTCTGAGGGTTGTTTT
Human *IL-1*	Forward	GCTCGCCAGTGAAATGATGG
	Reverse	GGTGGTCGGAGATTCGTAGC
Human *CCN2*	Forward	TGGCCCAGACCCAACTATGA
	Reverse	TTGGTAACCCGGGTGGAGAT
Human *EGR2*	Forward	ACCGCCTCCTCCTCCTTATT
	Reverse	GGGTAGGCCAGAGAGGAAGA
Human *NR4A1*	Forward	CCCTGAAGTTGTTCCCCTCAC
	Reverse	GCCCTCAAGGTGTGGAGAAG
Human *GAPDH*	Forward	GGGCTCTCCAGAACATCATCC
	Reverse	GGTCCACCACTGACACGTTG
Mus *Srebp1*	Forward	TCATCAACAACCAAGACAGT
	Reverse	GCCAGAGAAGCAGAAGAG
Mus *Fabp1*	Forward	GTCAAGGCAGTCGTCAAG
	Reverse	TGGTATTGGTGATTGTGTCT
Mus *Acc1*	Forward	AGCAGTTACACCACATACAT
	Reverse	TACCTCAATCTCAGCATAGC
Mus *Il-1*	Forward	CTTCAGGCAGGCAGTATC
	Reverse	CAGCAGGTTATCATCATCATC
Mus *Ccl-2*	Forward	CAATGAGTAGGCTGGAGAG
	Reverse	GAAGTGCTTGAGGTGGTT
Mus *Tnf-α*	Forward	GTGGAACTGGCAGAAGAG

	Reverse	GCTACAGGCTTGTCACTC
Mus *Gapdh*	Forward	CTCTGGAAAGCTGTGGCGTGATG
	Reverse	ATGCCAGTGAGCTTCCCGTTCAG
siNC		UUCUCCGAACGUGUCACGU
siEGR2_1		UGACAUGACUGGAGAGAAG
siEGR2_2		GAGAAGAGGUCGUUGGAUC
siEGR2_3		CCAGAAGGCAUAAUCAAUA

Table S2 Primers for plasmid construction

Gene	Species	F/R	Primer sequence (5'-3')
pLV CMV -CCN2-Myc	Homo	F	GTACCGAGCTCGGATCCatgaccgccgccagtatggg
		R	TCTGAGATGAGTTTTTGTTCtgccatgtctccgtacatcttc c
PLV CMV -NR4A1-Myc	Homo	F	GTACCGAGCTCGGATCCatgccctgtatccaagcccaatatg g
		R	TCTGAGATGAGTTTTTGTTCgaagggcagcgtgtccatga

Table S3 Antibody

Antibody	Cat No.	Manufacturer	MW (kDa)
Anti-β-actin mAb	4967	Cell Signaling Technology	45
Anti- CD36 mAb	A26251	ABclonal	70
Anti- Fatty Acid Synthase (FASN) Rabbit mAb	A19050	ABclonal	273
Anti- SCD1 Rabbit mAb	A26246	ABclonal	42
Anti- FABP1 Rabbit mAb	A11213	ABclonal	14
Anti- CPT1A Rabbit mAb	A27657	ABclonal	88
Anti- ACC1 Rabbit mAb	A19627	ABclonal	266
Anti- ACSL1 Rabbit mAb	A26078	ABclonal	78
Anti- PPARα Rabbit mAb	A25296	ABclonal	52

Anti- DGAT2 Rabbit Polyclonal Antibody	ER60651	HUABIO	42
Anti- PCSK9 (D5K4S) Rabbit Monoclonal Antibody	55728	cell Signaling Technology	65
Anti- Anti-Perilipin 2 Antibody	ab108323	Abcam	48
Anti- HRP-conjugated Goat anti-Rabbit IgG (H+L)	AS014	ABclonal	-

Supplement figures

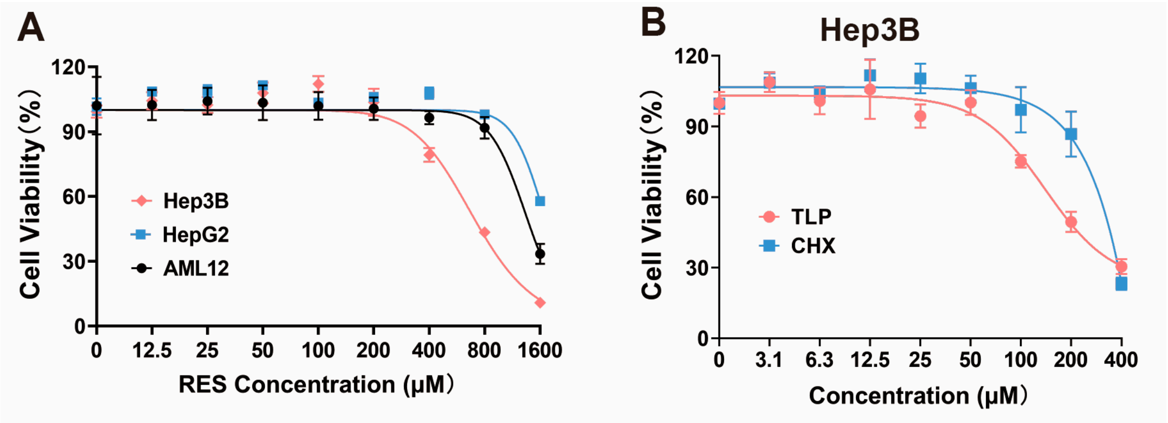

Fig S1. Fucoxanthin ameliorates metabolic dysfunction-associated steatohepatitis via suppression of CD36-driven fatty acid uptake. (A) Cytotoxicity of RES in Hep3B, HepG2 and AML12 cells were assessed by CCK-8 assay following 48-hour exposure. **(B)** Cell viability of Hep3B cells treated with TLP and CHX for 48 hours.

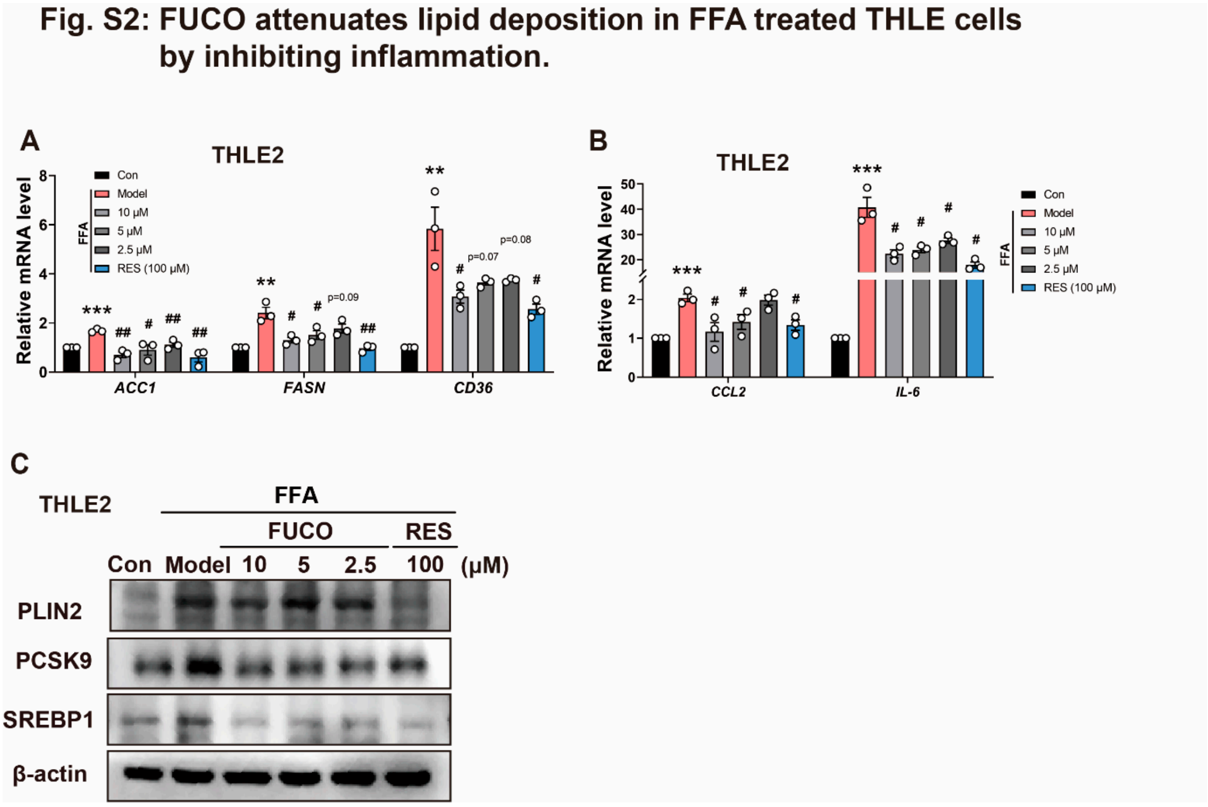

Fig. S2. FUCO attenuates lipid deposition in FFA-treated THLE2 cells by inhibiting inflammation.(**A, B**) THLE2 cells were treated with free fatty acids (FFA, model group) and then exposed to different concentrations of FUCO (10, 5, and 2.5 μM) or Resmetirom (RES, 100 μg/mL). The mRNA levels of lipid metabolism-related genes, including ACC1, FASN, and CD36 (**A**), and inflammation-related genes, including CCL2 and IL-6 (**B**), were determined by qRT-PCR.(**C**) Protein expression levels of PLIN2, PCSK9, and SREBP1 in THLE2 cells after the indicated treatments were analyzed by Western blotting. β-actin was used as the loading control.Data are presented as the mean ± SEM. **P* < 0.05, ***P* < 0.01, ****P* < 0.001 vs. con; #*P* < 0.05, ##*P* < 0.01 vs. model group.

Fig. S3: EGR2 is involved in FUCO-mediated suppression of CD36 expression and lipid accumulation in THLE2 cells

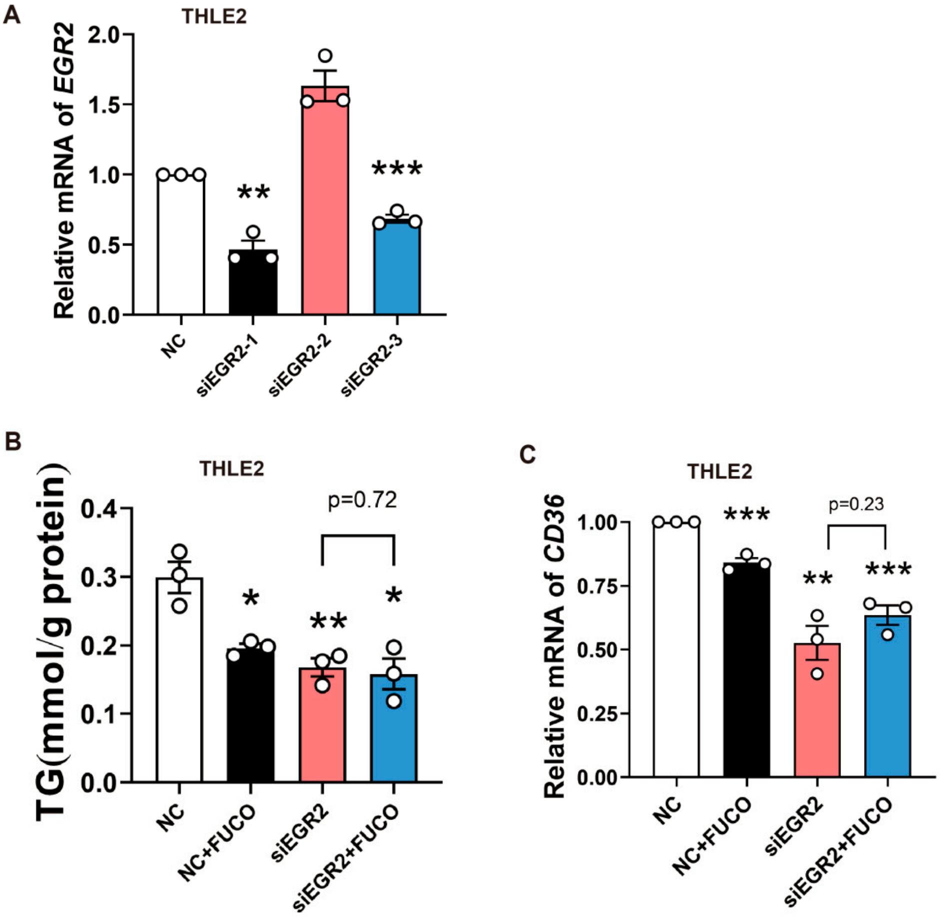

Fig. S3. EGR2 is involved in FUCO-mediated suppression of CD36 expression and lipid accumulation in THLE2 cells. (**A**) THLE2 cells were transfected with three independent siRNAs targeting EGR2 (siEGR2-1, siEGR2-2, and siEGR2-3), and EGR2 mRNA expression was measured by qRT-PCR to determine knockdown efficiency.(**B**) THLE2 cells were treated with NC, NC + FUCO (10 μM), siEGR2, or siEGR2 + FUCO (10 μM), and intracellular triglyceride (TG) content was measured and normalized to total protein.(**C**) CD36 mRNA expression in THLE2 cells under the indicated treatments was analyzed by qRT-PCR. Data are presented as the mean ± SEM. Each dot represents one independent biological replicate. * $p< 0.05$, **$p < 0.01$, ***$p < 0.001$ vs. NC; p values are indicated above the brackets.
